# Microbial Source Tracking and Community Assembly Mechanisms in Fenhe Reservoir Wetland

**DOI:** 10.3390/microorganisms14061225

**Published:** 2026-05-29

**Authors:** Xue Wang, Jinxian Liu, Baofeng Chai, Rui Yang

**Affiliations:** 1 Shanxi Key Laboratory for Ecological Restoration of Loess Plateau, Institute of Loess Plateau, Shanxi University, Taiyuan 030006, China; 202113202005@email.sxu.edu.cn (X.W.); liujinxian@sxu.edu.cn (J.L.); 2 School of Environment and Resources, Taiyuan University of Science and Technology, Taiyuan 030024, China

**Keywords:** reservoir wetland, microbial community assembly, source tracking

## Abstract

The structure and assembly of microbial communities are fundamental to regulating biogeochemical processes and maintaining water quality in reservoir ecosystems. In this study, Fenhe Reservoir, a reservoir wetland, and Gonghai Lake, a natural lake, were selected to investigate microbial community sources and assembly mechanisms using high-throughput sequencing combined with source tracking and phylogenetic null modeling. The results demonstrate pronounced spatial variation in both microbial sources and community assembly processes within the reservoir. In the upstream region of reservoir, microbial communities were shaped by riverine inputs, with variable selection as the main assembly driver. In downstream zones, homogeneous dispersal was identified as the predominant assembly mechanism, yielding community structures analogous to those typically observed in the natural lakes, wherein microbial sources were primarily restricted to shoreline sediments and the overlying water column. These findings highlight the unique dual structure of artificial reservoir microbial dynamics: upstream regions dominated by external inputs and strong selection pressures, and downstream zones driven by internal dispersal and localized processes.

## 1. Introduction

Reservoir wetland ecosystems play a vital role in maintaining biodiversity, regulating hydrological processes, and providing key ecological services [1]. Microbial communities, as fundamental and core biological components of reservoir wetlands, are irreplaceable in structuring food webs and sustaining nutrient cycling [2,3]. By driving the biogeochemical cycling of key elements such as carbon and nitrogen, microbes participate in crucial processes, including organic matter decomposition, nutrient regeneration, and pollutant degradation. Moreover, due to their high sensitivity to environmental disturbances, microbial community structures serve as reliable bioindicators for assessing the ecological health of reservoir wetland ecosystems. Therefore, gaining a deeper understanding of the mechanisms shaping microbial community assembly in reservoir wetlands not only has scientific significance for elucidating ecosystem functions but also provides an important basis for wetland conservation and restoration.

In reservoir wetland ecosystems, microbial community assembly is influenced by upstream sources, environmental conditions, and microbial dispersal. Traditionally, rivers (lotic systems) and lakes (lentic systems) have been studied as independent ecosystems. However, recent research increasingly recognizes the river–lake system as a continuum, with mutual influences between upstream and downstream environments [4]. Rivers connect terrestrial and aquatic ecosystems, transporting large quantities of microorganisms downstream. This upstream input, known as the “mass effect,” can significantly affect downstream microbial communities [5]. In lentic habitats, lateral inputs from the surrounding land are also potential sources of community assembly. Soil and vegetation along the wetland shoreline can contribute microbes to the waterbody through surface runoff, subsurface flow, or biotic vectors, resulting in microbial exchange between terrestrial and aquatic habitats [5]. In addition, atmospheric deposition, including airborne dust and precipitation, can introduce microbes from distant sources into wetland waters. Although their impact may be relatively limited, they should not be overlooked [6]. In reservoir wetlands, it remains unclear whether microbial community assembly is primarily driven by upstream inputs or by lateral diffusion and atmospheric deposition. Resolving this question is of great importance for reservoir ecosystem management, particularly for ensuring the safety of drinking water supplies.

The assembly of microbial communities is jointly influenced by deterministic and stochastic processes. However, due to strong system dependence, their relative contributions in reservoir wetland ecosystems remain unclear. Microbial inputs in reservoir wetlands originate from multiple sources, including upstream inflow, lateral inputs from surrounding terrestrial habitats, and atmospheric deposition, while environmental gradients and hydrodynamic conditions further regulate community structure. These drivers may either strengthen or weaken the roles of deterministic and stochastic processes, thereby contributing to inconsistencies among previous studies. Tang et al. [4] reported that in the Bosten Lake–Kaidu River continuum, pronounced environmental gradients—such as water salinity and suspended particles—enhanced environmental filtering, with pure environmental and spatial factors explaining 13.7% and 5.6% of community variation, respectively. In contrast, Yu et al. [7] found that in a closed lake with limited external inputs, both particle-associated and free-living bacterial communities were mainly shaped by deterministic processes, indicating a dominant role of environmental filtering under relatively stable conditions. Conversely, Wang et al. [8] observed that stochastic processes dominated microbial community assembly in both open and closed lakes, although community similarity remained significantly correlated with physicochemical parameters, suggesting that environmental constraints persist even under high-dispersal or dynamic conditions. Additional evidence was provided by Liu et al. [9], who found pronounced phylogenetic overdispersion in the Jinze Reservoir, where temperature and NH_4_^+^–N jointly regulated shifts between stochastic and deterministic processes, and spatial variation was primarily driven by dispersal limitation (61.68%) and heterogeneous selection (26.65%). Similarly, Wang et al. [10] showed that in the deep, oligotrophic reservoir, microbial community assembly across water layers was also predominantly stochastic, with the neutral model explaining 51.9–63.7% of community variation, surface communities exhibiting the highest migration rates, and fungal taxa showing the strongest diffusion limitation. Overall, these studies indicate that the dominance of deterministic versus stochastic processes in reservoir wetlands is highly dependent on hydrological context, external input intensity, and environmental gradients and therefore remains a key scientific question requiring further investigation.

In this study, the Fenhe (FH) Reservoir (an open reservoir wetland ecosystem) was selected as the research site, with Gonghai (GH) lake (a natural wetland ecosystem) serving as a reference. By integrating microbial source tracking and community assembly analysis, the study aims to address the following scientific questions: (1) In reservoir wetland ecosystems, are microbial communities primarily assembled from upstream water inputs, or do lateral diffusion and atmospheric deposition play a dominant role? (2) Do microbial community assembly mechanisms (deterministic vs. stochastic) differ significantly between artificial and natural aquatic systems? Clarifying the main sources and assembly mechanisms of microbial communities in reservoir wetland ecosystems is crucial for maintaining ecosystem stability, enhancing water purification capacity, and ensuring drinking water safety. This will not only contribute to scientifically evaluating the potential risks of exogenous microbial inputs to aquatic microecology but also provide theoretical and practical guidance for precisely managing microbial structures in water sources and preventing water quality anomalies and off-flavor compound formation.

## 2. Material and Methods

### 2.1. Site Description, Sampling Procedures, and Physicochemical Property Analysis

**Figure 1:**
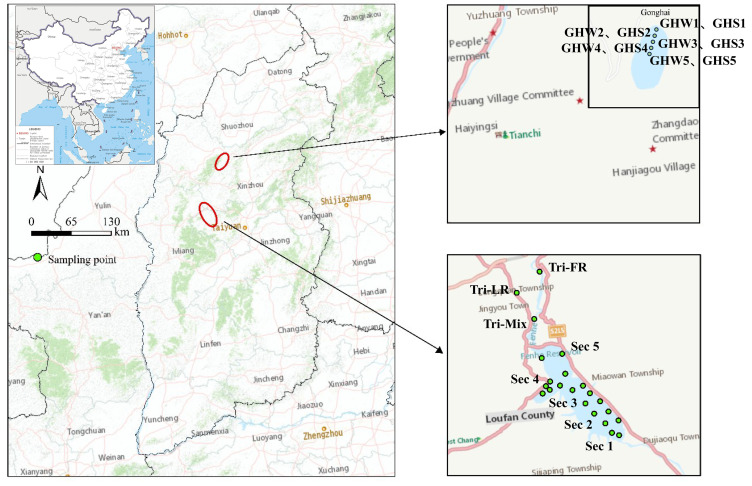
Location of study sites and distribution of sampling points. The inset map (upper left) indicates the geographic location of Shanxi Province, China, with the study area highlighted. The red circle in the main map indicates the location of the Fenhe Reservoir and GH Lake within Shanxi Province. Green dots represent the sampling sites distributed across different functional zones (inflow, transition, and lacustrine zones) of the reservoir. Schematic diagram of the Fenhe Reservoir showing the locations of sampling sites. Sec1–Sec5 represent different sections within the Fenhe Reservoir. Tri-FR and Tri-LR indicate tributary transects, while Tri-Mix represents the mixing zone of the two tributaries. Schematic diagram of the GH Lake included sediment samples (GHS1–GHS5) and overlying water samples (GHW1–GHW5) collected along a shore-to-center transect. GH0–GH10 represent vertical water samples collected at different depths in the center of GH Lake.

This study conducted sampling in two wetlands, an artificial reservoir and a natural lake. The artificial reservoir includes the FH Reservoir and upstream tributaries, which are in Loufan County, Taiyuan City, Shanxi Province (111°89′17″ E, 38°08′92″ N) (Figure 1). The region features a northern temperate continental monsoon climate with an annual precipitation of 428 mm. It is 15 km long from north to south and 5 km wide from east to west, with a total area of 32 km^2^ and an average depth of 6.50 m. The study area was divided into three regions based on the physical and chemical characteristics and ecological functions of the water body, namely the upstream confluence area, shallow water area, and deep-water area. Three transects have been set up for upstream water respectively, namely the water sample of FH River (Tri-FR), Lanhe River (Tri-LR), and the mixing point of them (Tri-Mix), as well as the sediment samples corresponding to each sampling point (Tri-S-FR, Tri-S-LR, and Tri-S-Mix). In the FH Reservoir, the sampling sites were divided into five zones, designated as Sec1, Sec2, Sec3, Sec4, and Sec5. We also collected the overlying water and sediment on the shore corresponding to each transect sampling point. In shallow water area, we collected 8 water samples and 4 sediment samples along the river channel. Five replicate samples were collected at each sampling site. Given that microbial diversity and metabolic activity are relatively high in summer reservoirs of northern China, this period provides an optimal window for capturing microbial community composition and assembly characteristics across distinct functional zones. Accordingly, we conducted field sampling in July 2022, collecting 170 water samples from 34 spatially distributed sites.

The natural lake is in GH Lake, Ningwu County, Shanxi Province. The region belongs to the warm temperate continental monsoon climate with an average annual temperature of 6.2 °C and an average annual rainfall of 462.5 mm, of which summer (June to August) precipitation accounts for more than 65%. We collected sediment (GHS1 to GHS5) and overlying water (GHW1 to GHW5) samples from the shore to the center of the lake and vertically collected water samples every 2 m in the center of the lake (GH0, GH2, GH4, GH6, GH8, GH10).

The sediment cores were collected using a PVC sampling corer (height: 20 cm; diam.: 4 cm). Overlying water samples were collected from the overlying water at a height of 0–10 cm above the sampling point for sediment core. The samples were immediately transported to the laboratory after collection on the same day.

### 2.2. Physicochemical Property Analysis

The following physicochemical factors were measured according to previously described methods [11,12]. Environmental properties such as water pH, dissolved oxygen (DO), electrical conductivity (EC), salinity (SAL), ammonium (NH_4_^+^-N), and nitrate (NO_3_^−^-N) content were monitored at the sampling site using a portable mul-tiparameter water monitoring probe (Aquaread AP-5000, Kent County, UK). Total organic carbon (TOC) and total carbon (TC) were analyzed using a TOC analyzer (Shimadzu, TOC-_VCPH_, Shimane, Kyoto, Japan).

### 2.3. DNA Extraction, PCR Amplification, and Illumina MiSeq Sequencing

Microorganisms in water samples were collected by filtration through a 0.2 μm pore size membrane filter (Millipore, Jinteng, China). The biomass-containing filters were cut into pieces, placed into centrifuge tubes, and subjected to DNA extraction by MagaBio Soil/Feces Genomic DNA Purification Kit (Hangzhou Bioer Technology Co. Ltd., Hangzhou, China). DNA concentration and purity were determined using a NanoDrop ND-2000 spectrophotometer (Thermo Fisher Scientific Inc., Waltham, MA, USA), and DNA integrity was assessed by 1% agarose gel electrophoresis [11].

The primers 338F (5′-ACTCCTACGGGAGGCAGCAG-3′) and 806R (5′-GGACTACHVGGGTWTCTAAT-3′) were used to amplify the V3–V4 hypervariable region of the bacterial 16S rRNA gene for characterization of bacterial community composition and structure [13]. For fungal community analysis, the internal transcribed spacer (ITS) region was amplified using the universal primer pair ITS1F (5′-CTTGGTCATTTAGAGGAAGTAA-3′) and ITS2 (5′-GCTGCGTTCTTCATCGATGC-3′) [14]. PCR amplification was performed in triplicate using a 20 μL reaction system containing 4.0 μL 5× FastPfu Buffer, 2.0 μL 2.5 mM dNTPs, 0.8 μL of each primer (5 μM), 0.4 μL FastPfu Polymerase, 0.2 μL BSA, and 10 ng template DNA, with ddH_2_O added to a final volume of 20 μL. PCR products were examined by 2% agarose gel electrophoresis, purified using an Axygen DNA Gel Extraction Kit (Axygen Biosciences, Union City, CA, USA), and quantified using a Quantus™ Fluorometer (Promega, Madison, WI, USA). Purified amplicons were pooled at equimolar concentrations and used for library preparation according to the standard protocol of the ALFA-SEQ DNA Library Prep Kit. Library fragment size distribution was assessed using the Qsep400 High Throughput Nucleic Acid and Protein Analysis System (Hangzhou Houze Biotechnology Co., Ltd., Hangzhou, China), and library concentration was quantified using a Qubit 4.0 fluorometer (Thermo Fisher Scientific, Waltham, MA, USA). Sequencing adapters and sample-specific index barcodes were added during library construction, followed by paired-end sequencing on an Illumina MiSeq platform (Guangdong Magigene Biotechnology Co., Ltd., Shenzhen, China).

Raw sequencing reads were subjected to quality control procedures, including the removal of low-quality reads, primer sequences, and chimeric sequences. Sequence processing was performed using QIIME2, and chimeric sequences were identified and removed using USEARCH. Given the substantial sequence heterogeneity and length variation of the fungal ITS region, high-quality sequences were clustered into operational taxonomic units (OTUs) at a 97% similarity threshold using UPARSE v7.0.1090. This approach reduced the potential influence of excessive sequence variability and provided a suitable framework for downstream analyses of microbial community composition, source tracking, and community assembly processes. Taxonomic annotation of bacterial and fungal OTUs was conducted against the SILVA (version 138.1) and UNITE (version 8.0) databases, respectively. For the 16S rRNA dataset, non-target and unclassified sequences were removed prior to downstream analyses to ensure accurate characterization of bacterial communities [15].

For the bacterial dataset, raw sequencing reads ranged from 105,402 to 131,972 per sample. Following quality filtering, more than 99% of reads were retained, yielding 92,566–112,808 high-quality tags per sample. Q20 and Q30 values ranged from 98.1–98.5% and 93.7–94.6%, respectively. For the fungal dataset, raw sequencing reads ranged from 60,328 to 92,399 per sample. More than 99% of reads were retained after filtering, yielding 44,117–85,252 high-quality tags per sample. Q20 and Q30 values ranged from 99.5–99.9% and 97.8–99.5%, respectively. To minimize biases associated with uneven sequencing depth among samples, bacterial and fungal datasets were rarefied to an even sequencing depth of 48,747 and 27,758 reads per sample, respectively, prior to downstream analyses.

It should be noted that the selected primers specifically targeted bacterial 16S rRNA and fungal ITS regions. Therefore, the microbial communities analyzed in this study mainly represent bacterial and fungal community composition rather than the total microbial community.

### 2.4. Statistical Analysis

Beta nearest taxon index (*β*NTI) was calculated in the R package ‘picante’ (v. 1.8.2) with the function ses.mntd based on the phylogenetic tree with 999 randomizations across all samples. Bray–Curtis dissimilarity (RCbray) was calculated in the R package ‘vegan’ (v. 2.6-4) and ‘parallel’ (v. 4.1.3) [12]. Based on this, integrating the *β*NTI and RCbray values to categorized assembly processes, including homogeneous selection (*β*NTI > 2), heterogeneous selection (*β*NTI < −2), dispersal limitation (|*β*NTI| < 2, RCBray > 0.95), homogenizing dispersal (|*β*NTI| < 2, RCBray < −0.95) and undominated (|*β*NTI| < 2, |RCBray| < 0.95) [16,17], we used Source Tracker (v.1.0), based on a Bayesian approach, to estimate the sources of the microbial communities [18]. In this analysis, upstream inputs (Tri-FR, Tri-LR), sediments, and overlying water were defined as potential source environments (Source), while microbial communities in reservoir water samples were treated as sinks (Sink). The model estimates the proportional contributions of each source to the sink communities based on differences in community composition. Default parameters were applied, including rarefaction to an even sequencing depth, 1000 Gibbs sampling iterations, and a burn-in of 100 iterations.

Differences in physicochemical factors were assessed through one-way ANOVA. Microbial diversity index was calculated using the R package ‘vegan’ (v. 4.1.3), and the effects of physicochemical factors on these indices of microbial diversity were estimated by mantel test analyses and visualized with heat maps [19].

## 3. Results and Discussion

### 3.1. Microbial Source Analysis in Wetland Ecosystems

**Figure 2:**
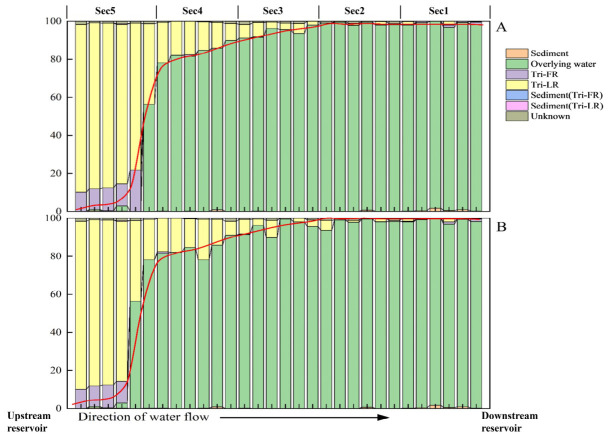
Source composition of bacterial (**A**) and fungal (**B**) communities across reservoir sections (Sec1–Sec5) and upstream transects (Tri-FR, Tri-LR).

The microbial inputs from upstream, shoreline, and sediments contribute to the high complexity of microbial sources in the reservoir. An analysis of microbial source contributions across various functional zones of the FH Reservoir revealed clear spatial differentiation between bacterial and fungal origins (Figure 2). In upstream zones (Sec5), microbial communities were almost entirely derived from riverine inputs, with bacterial and fungal contributions both reaching 98–99%, indicating strong exogenous control. Wu et al. [20] reported that upstream regions of tropical reservoirs, due to high watershed inflow intensity, often receive an abundance of allochthonous microbes, resulting in greater community complexity than in reservoir cores. Wang et al. [21] observed that microbial inputs from tributaries contribute significantly to microbial communities in adjacent downstream areas of the Three Gorges Reservoir. These results indicate that microbial communities in upstream zones are strongly influenced by exogenous inputs, with river-borne microbes dominating community composition. Exogenous microbial inputs and environmental pressures play a dominant role in shaping the microbial community structure in this region [22,23,24,25].

As water flows into the midstream and downstream sections of the reservoir, the contribution from riverine sources decreases markedly, with bacterial input falling to approximately 48% and fungal input to about 20%. Concurrently, non-point sources such as agricultural runoff and domestic wastewater become dominant, accounting for up to 99% of microbial inputs in the outlet region. At this location, bacterial and fungal contributions reached 99% and nearly 100%, respectively. Chen et al. [26] reported that significant structural differences in microbial communities between upstream and downstream regions of dammed sections along the Lancang River. Compared to the upstream, microbial communities in these two regions are primarily influenced by shoreline diffusion, likely due to reduced flow, increased retention time, and weakened hydrodynamic mixing [27,28,29].

**Figure 3:**
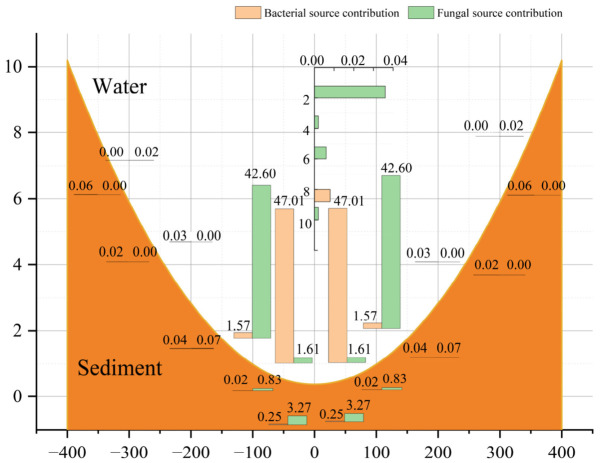
Source composition and spatial variation of bacterial and fungal communities in the natural wetland.

To further contrast microbial source mechanisms across ecosystem types, a natural lake—GH Lake—was selected as a reference. The results showed that microbial inputs were concentrated nearshore (Figure 3). For example, in sediment sample GHS5, fungal contributions reached 3.27%, while overlying water samples GHW4 and GHW5 contributed substantially to the central lake microbiota, at 43.60% and 47.01%, respectively. In contrast, central lake water samples (GH0–GH8) exhibited minimal microbial input, with average fungal and bacterial contributions below 0.05%. This pattern mirrors the bacterial source profile in the midstream and downstream zones of the FH Reservoir, suggesting that microbial communities in both systems are primarily driven by shoreline and sediment-derived inputs rather than by upstream water or pelagic microbial assemblages.

These findings suggest that microbial sources in reservoir wetlands can be broadly categorized into two types. In the upstream zones, microbial communities are largely shaped by riverine inputs, reflecting watershed characteristics and short-term hydrological transport [29,30]. In the downstream zones, where water flow slows, residence time increases, and hydrodynamic mixing weakens, microbial communities are more influenced by local retention, deposition, and shoreline diffusion [28,29]. These zones exhibit source patterns akin to those of natural lakes.

### 3.2. Microbial Community Assembly Mechanisms in Wetland Ecosystems

Disentangling the relative contributions of deterministic and stochastic processes in microbial community assembly is a key area of research, as these mechanisms play critical roles in shaping biodiversity and ecosystem function across wetland habitats [27,31]. Community assembly is influenced by multiple ecological processes, including environmental filtering, species dispersal, ecological drift, and historical contingency. The dominance of each process is often determined by spatial structure, hydrodynamic regimes, exogenous inputs, and disturbance intensity [32]. Understanding the mechanisms underlying microbial community assembly in reservoir wetlands is crucial for assessing ecosystem functioning and developing strategies for ecological protection [33].

**Figure 4:**
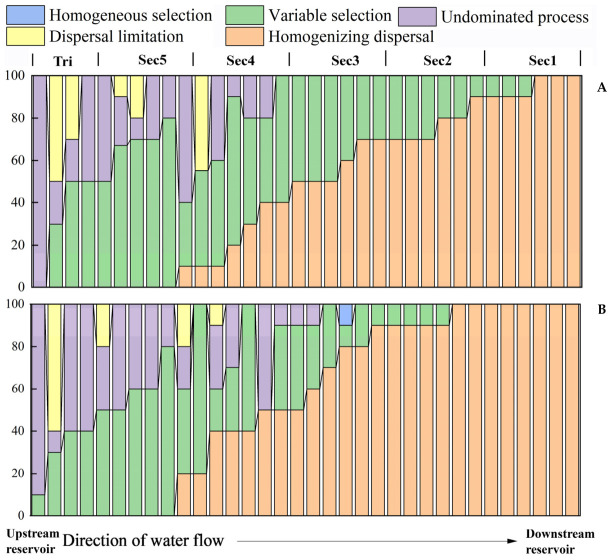
Community assembly of bacterial (**A**) and fungal (**B**) in the reservoir wetland.

In the FH Reservoir, microbial communities displayed distinct assembly patterns across different zones. In the tributary region (Tri-FR, Tri-LR and Tri-Mix), both bacterial and fungal communities were primarily shaped by variable selection and undominated processes (Figure 4). These areas are influenced by complex inputs from catchment runoff and non-point pollution, resulting in high variability in community composition. Consequently, variable selection played a dominant role in community assembly [34]. In regions impacted by agricultural runoff and domestic wastewater, microbial diversity tends to decline, with enrichment of specific functional genes (e.g., nitrate reduction and organic degradation genes), further reflecting the role of environmental pressures in shaping microbial assemblages [22,23,24,25].

In the upper reservoir region (Sec5), where tributaries connect directly to the main reservoir, community assembly was also dominated by environmental selection and undominated processes. Phylogenetic clustering results showed that the community composition in Sec5 was clearly separated from that in the downstream sections of the reservoir (Sec1 to Sec4), indicating a strong influence of exogenous riverine inputs (Figure S1). Notably, Verrucomicrobia and Chytridiomycota were significantly enriched in Sec5 (Figure S2). These phyla are frequently associated with the degradation of complex organic matter and terrestrial organic inputs, suggesting that the inflow region may receive substantial amounts of organic matter and nutrients transported by rivers. Previous studies also showed that carbon fixation and carbon degradation processes were more active in shallow water regions [27]. In particular, functional genes related to carbohydrate metabolism, polysaccharide degradation, and the Calvin Benson Bassham (CBB) cycle were significantly enriched in Sec5, further indicating that exogenous organic carbon inputs promoted microbially mediated carbon cycling processes in this region.

However, moving downstream, homogeneous dispersal became increasingly influential. Dispersal-driven processes accounted for over 90% to community assembly in Sec1, suggesting a more stable and uniform aquatic environment promoted the homogenization of microbial community [35]. Water environment in downstream reservoir areas is under relatively low environmental pressure, with microbial communities more strongly shaped by local conditions than by upstream hydrological dynamics [27]. This transition indicates that hydrological connectivity and environmental gradients jointly shaped microbial community assembly in the reservoir.

**Figure 5:**
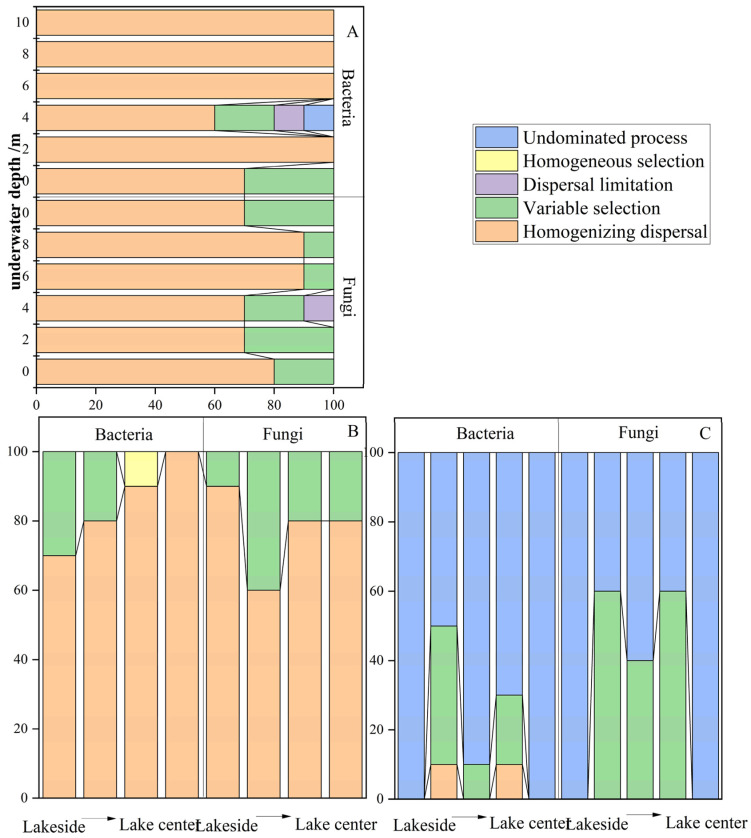
Community assembly of bacteria and fungi in the natural wetland system: (**A**) vertical depth, (**B**) overlying water, and (**C**) sediments.

For comparison with natural systems, GH Lake was used as a reference site. Sediment samples (e.g., GHS3 and GHS4) exhibited high levels of undominated (stochastic) processes, indicating that community structure in these areas is influenced by historical sedimentation and ecological drift (Figure 5). In contrast, overlying water samples (e.g., GHW2 and GHW3) showed communities primarily structured by homogeneous dispersal, suggesting that weak environmental filtering and high dispersal rates govern microbial distribution. These patterns closely resemble those observed in the midstream and downstream zones of the FH Reservoir, highlighting convergent community assembly mechanisms between artificial and natural wetlands.

At a broader spatial scale, this upstream-to-downstream shift in community assembly mechanisms reveals the underlying logic of microbial biogeographic pattern formation in reservoir wetlands. In upstream regions, microbial communities are primarily governed by watershed-scale external drivers, including catchment geomorphology, land-use types, hydrological regimes, and exogenous pollutant inputs [30]. In addition, eutrophication-driven gradients in total carbon and nutrients play an important role in shaping spatial distribution patterns [36]. In midstream and downstream regions, microbial communities increasingly depend on local environmental constraints and internal selection processes [28,29]. Factors such as water residence time, hydrodynamic conditions, depth gradients, dissolved oxygen levels, temperature, and sediment physicochemical properties are closely associated with community assembly. In the absence of large-scale external pollutant inputs or acute contamination events, improvements in midstream and downstream water quality largely rely on the optimization of local environmental conditions, whereas the direct downstream propagation of upstream water quality improvements remains relatively limited [5].

### 3.3. Environmental Gradients Driving Microbial Community Patterns

The structure of microbial sources and community assembly mechanisms in the FH Reservoir exhibited clear changes along the direction of water transport. Significant differences in environmental conditions were observed among sampling sites (*p* < 0.001) (Table 1), highlighting a pronounced environmental gradient across the reservoir. In the inflow region, the water was characterized by high nutrient concentrations. For example, NO_3_^−^-N concentrations at Tri-LR and Tri-FR reached 14.0 mg/L and 10. 7 mg/L, respectively, while NH_4_^+^-N concentrations were 0.2 mg/L and 0.2 mg/L. Total carbon was also relatively high, reaching up to 47.1 mg/L. In addition, elevated electrical conductivity (up to 1917 μS/cm) and salinity further indicated strong watershed inputs in this region. Under these environmental conditions, large quantities of exogenous microorganisms entered the reservoir with river inflow, resulting in river-derived microbes dominating the community composition. Meanwhile, the high nutrient availability strengthened environmental filtering effects on community structure, which is consistent with the source patterns and community assembly processes described above.

As water flowed into the shallow zone of the reservoir, environmental conditions changed markedly. In this region, pH and dissolved oxygen increased (with DO reaching up to 10.2 mg/L), whereas nitrate concentrations decreased substantially (down to 3.0 mg/L). These patterns suggest that the shallow zone plays an important role in nutrient transformation and ecological purification. Stronger light availability and active primary production allow aquatic plants, algae, and attached microorganisms to assimilate and transform nutrients, thereby reducing nitrogen concentrations in the water column. At the same time, particle settling and microbial decomposition become more pronounced, gradually shifting the system from the highly disturbed conditions of the inflow area toward a more stable environment.

Further downstream, physicochemical parameters at the deep-water sampling sites became relatively stable. Dissolved oxygen remained between 9.5 and 9.8 mg/L, and pH ranged from 8.4 to 8.6, while nitrogen and organic matter concentrations decreased further. This indicates that after nutrient transformation in the shallow zone, the water body gradually reached a relatively balanced state. Under conditions of reduced environmental variability and weaker hydrodynamic disturbance, microorganisms can disperse more easily among sampling sites, resulting in more homogeneous community structures. Meanwhile, as the influence of river inputs diminishes, shoreline diffusion and sediment release become increasingly important microbial sources. These patterns are consistent with the source transitions shown in Section 3.1 and help explain the dominance of homogeneous dispersal in the downstream area described in Section 3.2.

**Figure 6:**
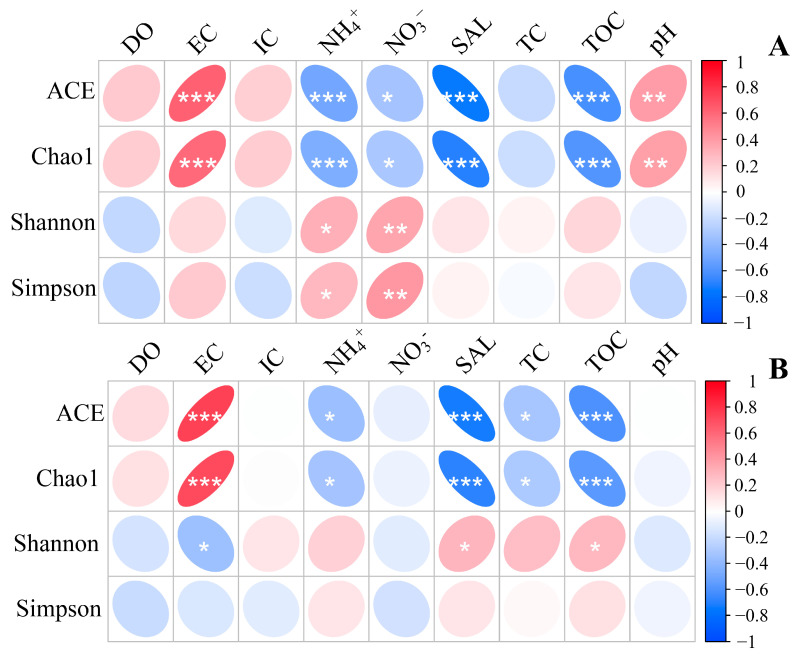
Relationships between environmental variables and the α-diversity of bacterial (**A**) and fungal (**B**) communities based on Spearman correlation analysis. * represent the degree of significance [*p* < 0.001 (***), *p* < 0.01 (**), *p* < 0.05 (*)].

Correlation analyses further revealed the potential environmental drivers of microbial diversity (Figure 6). Spearman correlation analysis showed that in planktonic fungal communities, the richness indices Chao1 and ACE were significantly positively correlated with electrical conductivity (EC) (*p* < 0.05), but negatively correlated with NH_4_^+^-N, salinity (SAL), total carbon (TC), and total organic carbon (TOC). These results suggest that elevated nutrient and organic matter loads may suppress fungal community richness [37]. In addition, the Shannon index showed a weak positive relationship with SAL and TOC, whereas no significant correlation was detected for the Simpson index, indicating that different diversity metrics respond differently to environmental variation.

In contrast, planktonic bacterial communities exhibited different response patterns. Spearman analysis indicated that the Shannon and Simpson indices were positively correlated with NH_4_^+^-N and NO_3_^−^-N concentrations (*p* < 0.05), suggesting that nitrogen availability is an important factor maintaining species evenness and structural stability within bacterial communities. This finding supports the applicability of the resource control hypothesis in wetland bacterial systems, whereby moderate nitrogen inputs can enhance diversity by increasing resource availability [38]. However, the richness indices Chao1 and ACE were positively correlated with EC and pH but negatively correlated with NH_4_^+^-N, NO_3_^−^-N, SAL, and TOC. These results suggest that under high nutrient loading, excessive external inputs of organic matter and salts may lead to niche compression, suppressing rare and environmentally sensitive taxa and ultimately driving community homogenization. This observation is consistent with previous studies showing that eutrophication can simplify microbial community structure and reduce ecosystem functional diversity in wetland environments [39].

Overall, high nutrient inputs in the river inflow region provide abundant resources for microbial growth, but excessive enrichment may also lead to simplified community structures. As water flows toward the interior of the reservoir, nutrient concentrations decline and environmental conditions become more stable, resulting in more homogeneous microbial communities. Therefore, nutrient availability, organic matter content, and ionic conditions likely represent key factors shaping the spatial patterns of microbial communities in this artificial reservoir ecosystem.

## 4. Conclusions

This study investigated the sources and community assembly mechanisms of microorganisms across different functional zones of the FH Reservoir and compared them with those of a natural lake ecosystem. The results reveal pronounced spatial heterogeneity in microbial source pathways and divergent community assembly processes within reservoir wetland ecosystems. The key findings are as follows:

(a) Microbial sources exhibit clear spatial differentiation. In the upstream zones, microbial communities are predominantly influenced by riverine inputs, reflecting strong allochthonous control. In contrast, the midstream and downstream zones are primarily shaped by lateral inputs from riparian zones and sediment release, with diminishing contributions from external sources.
(b) Community assembly mechanisms shift along the hydrological gradient. In the upstream areas, community assembly is mainly governed by variable selection and undominated processes, suggesting strong environmental filtering and external influence. Toward the midstream and downstream regions, homogeneous dispersal becomes the dominant mechanism, indicating a more stable and uniform aquatic environment where dispersal limitation is reduced.
(c) Microbial assembly patterns in the downstream reservoir are like those in natural lakes, with microbial communities primarily sourced from surrounding sediments and shoreline diffusion and shaped predominantly by homogeneous dispersal. This suggests that under certain hydrodynamic conditions, artificial aquatic systems may exhibit ecological processes comparable to those of natural wetlands.

## Figures and Tables

**Table 1:** Environmental factors among the samples. Values are shown with means ± standard error. Different letters within a row means significant difference among different samples (*p* < 0.001).

Varibles	pH	DO (mg/L)	EC (uS/cm)	SAL (ng/L)	NO_3_^−^-N (mg/L)	NH_4_^+^-N (mg/L)	TC (mg/L)	TOC (mg/L)
Tri-FR	7.7 ± 0.1 ^d^	6.6 ± 0.0 ^c^	1917 ± 4 ^a^	0.9 ± 0.0 ^a^	10.7 ± 0.6 ^b^	0.2 ± 0.0 ^b^	37.9 ± 0.1 ^b^	2.7 ± 0.1 ^e^
Tri-LR	7.8 ± 0.1 ^d^	7.8 ± 0.0 ^bc^	1409 ± 4 ^d^	0.7 ± 0.0 ^d^	14.0 ± 0.2 ^a^	0.2 ± 0.0 ^a^	47.1 ± 0.1 ^a^	3.2 ± 0.1 ^d^
Tri-Mix	7.8 ± 0.0 ^d^	6.6 ± 0.0 ^c^	1789 ± 8 ^b^	0.9 ± 0.0 ^b^	10.8 ± 0.1 ^b^	0.1 ± 0.0 ^bcd^	37.1 ± 0.1 ^b^	3.0 ± 0.1 ^de^
Sec5	8.1 ± 0.1 ^c^	10.2 ± 0.7 ^a^	1750 ± 28 ^bc^	0.9 ± 0.0 ^bc^	6.1 ± 0.3 ^c^	0.2 ± 0.0 ^a^	37.4 ± 1.1 ^b^	5.5 ± 1.2 ^a^
Sec4	8.3 ± 0.1 ^bc^	8.9 ± 0.2 ^ab^	1719 ± 2 ^c^	0.9 ± 0.0 ^c^	3.0 ± 0.2 ^e^	0.2 ± 0.0 ^bc^	30.9 ± 0.4 ^d^	4.8 ± 0.3 ^b^
Sec3	8.4 ± 0.0 ^abc^	9.5 ± 0.0 ^a^	1769 ± 8 ^bc^	0.9 ± 0.0 ^bc^	4.1 ± 0.1 ^d^	0.1 ± 0.0 ^cd^	34.2 ± 0.5 ^c^	5.3 ± 0.4 ^ab^
Sec2	8.6 ± 0.0 ^a^	9.5 ± 0.0 ^a^	1811 ± 1 ^b^	0.9 ± 0.0 ^b^	5.4 ± 0.1 ^c^	0.1 ± 0.0 ^d^	32.5 ± 0.1 ^cd^	5.0 ± 0.2 ^b^
Sec1	8.4 ± 0.0 ^ab^	9.8 ± 0.0 ^a^	1796 ± 8 ^b^	0.9 ± 0.0 ^b^	5.3 ± 0.1 ^c^	0.1 ± 0.0 ^de^	31.3 ± 0.1 ^d^	4.2 ± 0.1 ^c^

## Data Availability

Raw data were deposited in the Sequence Read Archive of NCBI under the accession number PRJNA1257555 (https://dataview.ncbi.nlm.nih.gov/obect/PRJNA1257555?rviewer=sblpks9ukbq7miks4vbagvdrqv, assessed on 1 December 2025).

## Supplementary Materials

The following supporting information can be downloaded at: https://www.mdpi.com/article/10.3390/microorganisms14061225/s1, Figure S1: OTU-level phylogenetic trees of bacterial (A) and fungal (B) communities across different habitats. Sec1–Sec5 represent different reservoir sections, and Tri, Sediment, Overlying water, and SedimentTri indicate tributary water, sediment, overlying water, and tributary sediment samples, respectively; Figure S2: Relative abundances of dominant bacterial (A) and fungal (B) phyla across different habitats.

## References

[1] Kajan K., Osterholz H., Stegen J., Udovič M.G., Orlić S. (2023). Mechanisms shaping dissolved organic matter and microbial community in lake ecosystems. Water Res..

[2] Cao Q., Wang H., Chen X., Wang R., Liu J. (2017). Composition and distribution of microbial communities in natural river wetlands and corresponding constructed wetlands. Ecol. Eng..

[3] Wang M., Weng X., Zhang R., Yang L., Liu Y., Sui X. (2022). The diversity and composition of soil microbial community differ in three typical wetland types of the sanjiang plain, northeastern China. Sustainability.

[4] Tang X., Xie G., Shao K., Hu Y., Cai J., Bai C., Gong Y., Gao G. (2020). Contrast diversity patterns and processes of microbial community assembly in a river-lake continuum across a catchment scale in northwestern China. Environ. Microbiome.

[5] Stadler M., del Giorgio P.A. (2022). Terrestrial connectivity, upstream aquatic history and seasonality shape bacterial community assembly within a large boreal aquatic network. ISME J..

[6] Jones S.E., Newton R.J., McMahon K.D. (2008). Potential for atmospheric deposition of bacteria to influence bacterioplankton communities. FEMS Microbiol. Ecol..

[7] Yu B., Xie G., Shen Z., Shao K., Tang X. (2023). Spatiotemporal variations, assembly processes, and co-occurrence patterns of particle-attached and free-living bacteria in a large drinking water reservoir in China. Front. Microbiol..

[8] Wang S., Hu Y., Fan T., Fang W., Liu X., Xu L., Li B., Wei X. (2023). Microbial community structure and co-occurrence patterns in closed and open subsidence lake ecosystems. Water.

[9] Liu M., Tong J., Zhu H., Bai X.-H. (2020). Phylogenetic processes and key driving factors of bacterial communities in Jinze Reservoir. Environ. Sci..

[10] Wang W., Wang R., Li Y., Zhang P., Gao M., Cao Y., Fohrer N., Zhang Y., Li B.L. (2025). Cross-sectional-dependent microbial assembly and network stability: Bacteria sensitivity response was higher than eukaryotes and fungi in the Danjiangkou Reservoir. J. Environ. Manag..

[11] Liu J., Li C., Jing J., Zhao P., Luo Z., Cao M., Ma Z., Jia T., Chai B. (2018). Ecological patterns and adaptability of bacterial communities in alkaline copper mine drainage. Water Res..

[12] Wang X., Liu J., Ren J., Chai B. (2024). Biotic Interaction Underpins the Assembly Processes of the Bacterial Community Across the Sediment–Water Interface in a Subalpine Lake. Microorganisms.

[13] Zhu J.-T., Lin H., Wu X., Li Z.-W., Lin A.-Y. (2019). Metataxonomics of Internal Transcribed Spacer amplicons in cerebrospinal fluid for diagnosing and genotyping of cryptococcal meningitis. Chin. Med. J..

[14] Dai T., Wen D., Bates C.T., Wu L., Guo X., Liu S., Su Y., Lei J., Zhou J., Yang Y. (2022). Nutrient supply controls the linkage between species abundance and ecological interactions in marine bacterial communities. Nat. Commun..

[15] Wang X., Liu J., Chai B., Wu T. (2025). Protozoa-driven micro-food webs shaping carbon and nitrogen cycling in reservoir ecosystems. Environ. Microbiome.

[16] Zhou J., Ning D. (2017). Stochastic community assembly: Does it matter in microbial ecology?. Microbiol. Mol. Biol. Rev..

[17] Jiao S., Yang Y., Xu Y., Zhang J., Lu Y. (2020). Balance between community assembly processes mediates species coexistence in agricultural soil microbiomes across eastern China. ISME J..

[18] Knights D., Kuczynski J., Charlson E., Zaneveld J., Mozer M.C., Collman R.G., Bushman F.D., Knight R.T., Kelley S.T. (2011). Bayesian community-wide culture-independent microbial source tracking. Nat. Methods.

[19] Oksanen J., Blanchet F.G., Kindt R., Legendre P., Minchin P.R., O’Hara R.B., Simpson G.L., Sólymos P., Stevens M.H.H., Wagner H. vegan: Community Ecology Package. R Package, 2012. http://CRAN.R-project.org/package=vegan.

[20] Wu D., Zou Y., Xiao J., Mo L., Lek S., Chen B., Fu Q., Guo Z. (2024). The spatiotemporal variations of microbial community in relation to water quality in a tropical drinking water reservoir, Southmost China. Front. Microbiol..

[21] Wang S., Hou W., Jiang H., Huang L., Dong H., Chen S., Wang B., Chen Y., Lin B., Deng Y. (2021). Microbial diversity accumulates in a downstream direction in the Three Gorges Reservoir. J. Environ. Sci..

[22] Jackson C.R., Churchill P.F., Roden E.E. (2001). Successional changes in bacterial assemblage structure during epilithic biofilm development. Ecology.

[23] Dini-Andreote F., Stegen J.C., van Elsas J.D., Salles J.F. (2015). Disentangling mechanisms that mediate the balance between stochastic and deterministic processes in microbial succession. Proc. Natl. Acad. Sci. USA.

[24] Yu H., Zhong Q., Peng Y., Zheng X., Xiao F., Wu B., Yu X., Luo Z., Shu L., Wang C. (2022). Environmental filtering by pH and salinity jointly drives prokaryotic community assembly in coastal wetland sediments. Front. Mar. Sci..

[25] Zhang N., Chen K., Chen J., Ji W., Yang Z., Chen Z. (2024). Response characteristics and community assembly mechanisms of nirS-type denitrifiers in the alpine wetland under simulated precipitation conditions. Biology.

[26] Chen J., Wang P., Wang C., Wang X., Miao L., Liu S., Yuan Q. (2018). Bacterial communities in riparian sediments: A large-scale longitudinal distribution pattern and response to dam construction. Front. Microbiol..

[27] Huang L., Bai J., Wang J., Zhang G., Wang W., Wang X., Zhang L., Wang Y., Liu X., Cui B. (2022). Different stochastic processes regulate bacterial and fungal community assembly in estuarine wetland soils. Soil Biol. Biochem..

[28] Yao L., Wu J., Liu S., Xing H., Wang P., Gao W., Wu Z., Zhou Q. (2024). Distinct drivers of bacterial community assembly processes in riverine islands in the middle and lower reaches of the Yangtze River. Microbiol. Spectr..

[29] Niño-García J.P., Ruiz-González C., Del Giorgio P.A. (2016). Interactions between hydrology and water chemistry shape bacterioplankton biogeography across boreal freshwater networks. ISME J..

[30] Luo X., Xiang X., Huang G., Song X., Wang P., Yang Y., Fu K., Che R. (2020). Bacterial community structure upstream and downstream of cascade dams along the Lancang River in southwestern China. Environ. Sci. Pollut. Res..

[31] Powell J.R., Karunaratne S., Campbell C.D., Yao H., Robinson L., Singh B.K. (2015). Deterministic processes vary during community assembly for ecologically dissimilar taxa. Nat. Commun..

[32] Shang Y., Wang X., Wu X., Dou H., Wei Q., Wang Q., Liu G., Sun G., Wang L., Zhang H. (2024). Bacterial and fungal community structures in Hulun Lake are regulated by both stochastic processes and environmental factors. Microbiol. Spectr..

[33] Xu Z., Hu J., Xin X., Wen L., Cao X., Zhang R., Kou X., Liu D., Liu H., Wang L. (2024). Geographical and environmental distance differ in shaping biogeographic patterns of microbe diversity and network stability in lakeshore wetlands. Ecol. Indic..

[34] Tian W., Li Q., Luo Z., Wu C., Sun B., Zhao D., Chi S., Cui Z., Xu A., Song Z. (2024). Microbial community structure in a constructed wetland based on a recirculating aquaculture system: Exploring spatio-temporal variations and assembly mechanisms. Mar. Environ. Res..

[35] Li X., Meng Z., Chen K., Hu F., Liu L., Zhu T., Yang D. (2023). Comparing diversity patterns and processes of microbial community assembly in water column and sediment in Lake Wuchang, China. PeerJ.

[36] Yang H., Xiong X., Tai Y., Xiao L.-J., He D., Wu L., Zhou L., Ren L., Wu Q.L., Han B.-P. (2025). Sediment bacterial biogeography across reservoirs in the Hanjiang river basin, southern China: The predominant influence of eutrophication-induced carbon enrichment. Front. Microbiol..

[37] Geng M., Zhang W., Hu T., Wang R., Cheng X., Wang J. (2022). Eutrophication causes microbial community homogenization via modulating generalist species. Water Res..

[38] Bledsoe R.B., Goodwillie C., Peralta A.L. (2020). Long-term nutrient enrichment of an oligotroph-dominated wetland increases bacterial diversity in bulk soils and plant rhizospheres. mSphere.

[39] Deng W., Chen S., Chen S., Xing B., Chan Z., Zhang Y., Chen B., Chen G. (2024). Impacts of eutrophication on microbial community structure in sediment, seawater, and phyllosphere of seagrass ecosystems. Front. Microbiol..

