# Supplementary material for: Microbial Source Tracking and Community Assembly Mechanisms in Fenhe Reservoir Wetland
Source: Microorganisms. 2026 May 29;14(6):1225. doi: 10.3390/microorganisms14061225 (PMC13303715; doi:10.3390/microorganisms14061225)
Supplement: Supplementary file 1 [file microorganisms-14-01225-s001.zip › microorganisms-4273975-supplementary.pdf]

Supplementary Information

Microbial Source Tracking and Community Assembly Mechanisms in Fenhe Reservoir Wetland

Number of pages: 2
Number of figures: 2

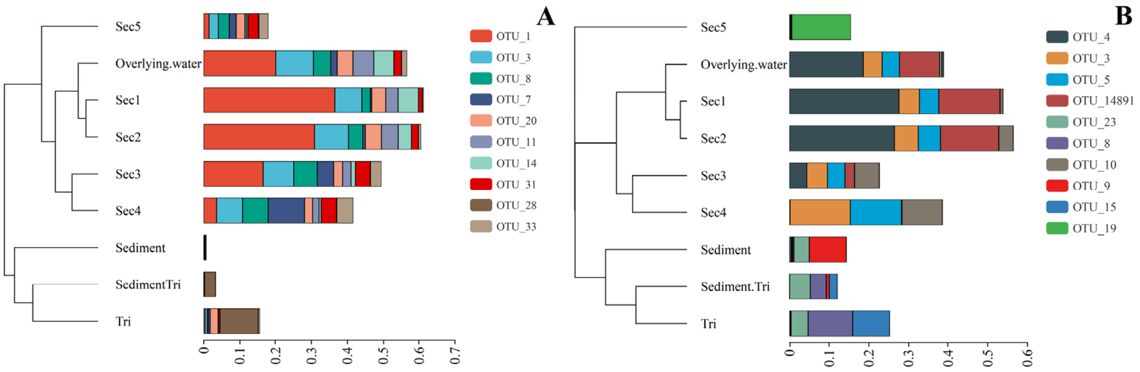

Figure S1. OTU-level phylogenetic trees of bacterial (A) and fungal (B) communities across different habitats. Sec1-Sec5 represent different reservoir sections, and Tri, Sediment, Overlying water, and SedimentTri indicate tributary water, sediment, overlying water, and tributary sediment samples, respectively.

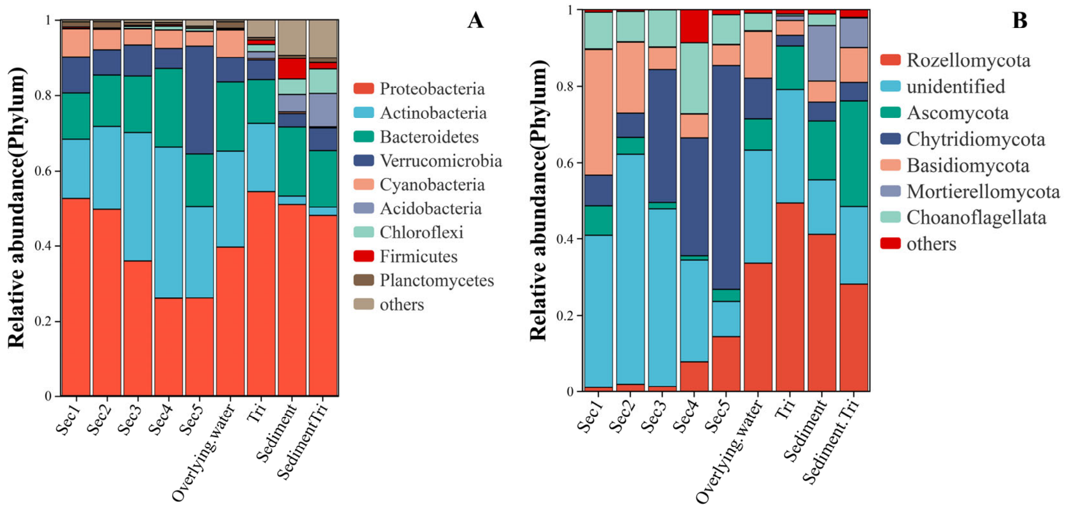

Figure S2. Relative abundances of dominant bacterial (A) and fungal (B) phyla across different habitats.
